# A new Schiff base 2-benzoylpyridine-based copper complex on boehmite nanoparticles as a recoverable nanocatalyst for the homoselective synthesis of 5-substituted tetrazoles†

**DOI:** 10.1039/d4ra03139c

**Published:** 2024-05-20

**Authors:** Elham Mohseni, Arash Ghorbani-Choghamarani, Bahman Tahmasbi, Masoomeh Norouzi

**Affiliations:** a Department of Chemistry, Faculty of Science, Ilam University P. O. Box 69315516 Ilam Iran b.tahmasbi@ilam.ac.ir; b Department of Organic Chemistry, Faculty of Chemistry, Bu-Ali Sina University Hamedan 6517838683 Iran a.ghorbani@basu.ac.ir

## Abstract

In this study, boehmite nanoparticles (B-NPs) were prepared by a simple process and then their surface was modified by (3-aminopropyl)triethoxysilane (3-APTES). The modified B-NPs (3-APTES@B-NPs) were functionalized by 2-benzoylpyridine Schiff-base ligand toward the immobilization of the Schiff-base 2-benzoylpyridine ligand on the 3-APTES@B-NPs's surface (2BP-Schiff-base@B-NPs). Finally, copper ions were coordinated with the supported Schiff-base ligand on B-NPs toward the formation of the final catalyst (Cu-2BP-Schiff-base@B-NPs). The prepared Cu-2BP-Schiff-base@B-NPs were characterized using FT-IR spectroscopy, BET analysis, XRD, SEM, AAS, TGA, EDX and elemental mapping. Further, Cu-2BP-Schiff-base@B-NPs were applied as a homoselective and recyclable catalyst for the synthesis of a diverse range of 5-substituted tetrazoles in PEG-400 as a green solvent. The main benefits of this protocol are high homoselectivity attributes, short reaction times, high product yields and TOF values, and further addition to the catalyst ability to be recycled at least four times without significantly losing catalytic efficiency.

## Abstract 2

In this research, the surface of boehmite NPs were modified and functionalized by 2-benzoylpyridine toward the immobilization of copper ions as a practical catalyst in the synthesis of tetrazoles.

## Graphical abstract

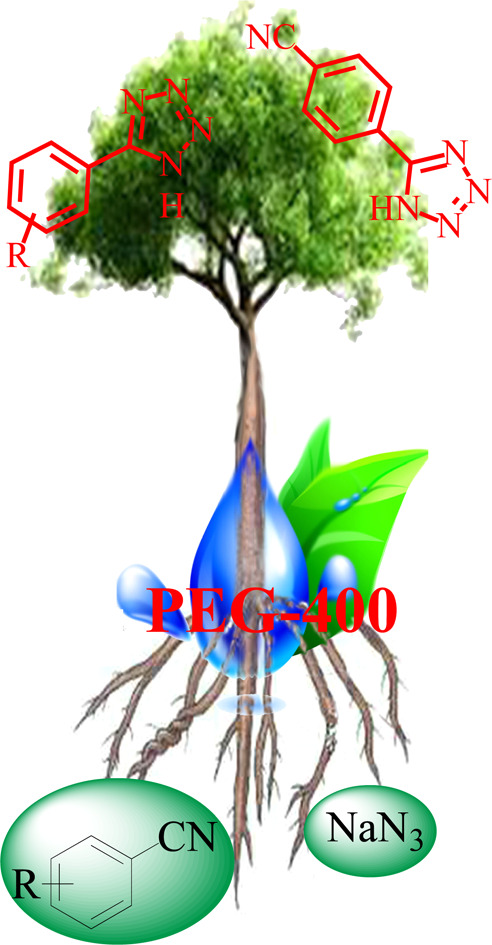

## 1. Introduction

Tetrazoles are a class of poly-aza-heterocyclic compounds that are not found in nature.^1,2^ Tetrazoles have recently received much attention due to their broad range of uses in medicine and biology, such as anticancer, antiviral, antiallergic, antibiotic, anti-HIV, and many other fields.^3–7^ The carbon–nitrogen ring system CN_4_H_2_ was firstly named by Bladin in 1885.^8,9^ In recent decades, green chemistry research has been focussed on new and efficient methods to prevent the production of chemical pollution and damage to the environment and earth using catalysts and green solvents.^10,11^ Homogeneous catalysts were mostly used in the synthesis of tetrazole derivatives due to their higher activity than their heterogeneous counterparts. Nevertheless, homogeneous catalysts suffer from various disadvantages, including difficulty in recycling and product contamination. To overcome the above-mentioned drawbacks, various strategies of heterogenized systems were devised, such as catalyst grafting on heterogeneous substrates *e.g.* cellulose^12^ and inorganic supports,^13–15^ and polymers.^16,17^ However, heterogeneous catalyst systems suffer from various disadvantages such as low catalytic performance. Given the disadvantages of homogenous and heterogeneous catalyst systems, researchers have put considerable effort into developing novel synthetic techniques to produce tetrazole derivatives utilizing nanostructured catalyst systems. Nanoparticles (NPs) are particles with dimensions less than 100 nanometers. Nanoparticles have been widely used in catalytic applications for the synthesis of various nanocatalysts. Since 2010, various nano-catalysts or catalysts on nanomaterials have been developed to mitigate drawbacks associated with the current methods for the synthesis of tetrazole derivatives.^4,18–28^ Boehmite nanoparticles (B-NPs) are one of the most available and cheap mineral compounds, which have recently been used as a support for catalysts, absorbents, flame retardants, ceramics, optical materials, and vaccine adjuvants, which are made from materials in a simple technique in the aqueous setting.^29^ Indeed, boehmite is one of the polymorph phases of aluminum oxide, also known as aluminum oxyhydroxide.^30–34^ It has a cubic orthorhombic structure of aluminum oxide hydroxide and can be provided in water utilizing available materials.^35^ In addition, boehmite has been used as a starting material for the synthesis of alumina and composite reinforcement material in ceramics, vaccine adjuvants, and cosmetics.^36^ Some unique properties of boehmite (*e.g.* high surface area, high density of hydroxyl groups in its surface and its high chemical/thermal stability) have made it useful as a catalyst support.^37–39^ Furthermore, boehmite nanoparticles are not air or moisture-sensitive, they can be prepared in water at room temperature.^40^ Hence, to keep up with the green chemistry principles, in this research, we designed, characterized, and synthesized the Cu-2BP-Schiff-base@B-NPs as a novel, efficient, and recyclable heterogeneous nanocatalyst to synthesize 5-substituted tetrazole derivatives.

## 2. Experimental

### 2.1. Preparation as Cu-2BP-Schiff-base@B-NPs nanocatalyst

For the preparation of B-NPs under vigorous stirring, 6.490 g of NaOH in 50 mL deionized water (DI-H_2_O) was added to the solution of Al(NO_3_)_3_·9H_2_O (20 g) in 30 mL DI-H_2_O, drop by drop. Then, the resultant suspension was dispersed in an ultrasonic bath for 3 h. The resultant boehmite NPs were filtered and washed with DI-H_2_O and baked in the oven at 220 °C for 4 hours.^41^

**Scheme 1:**
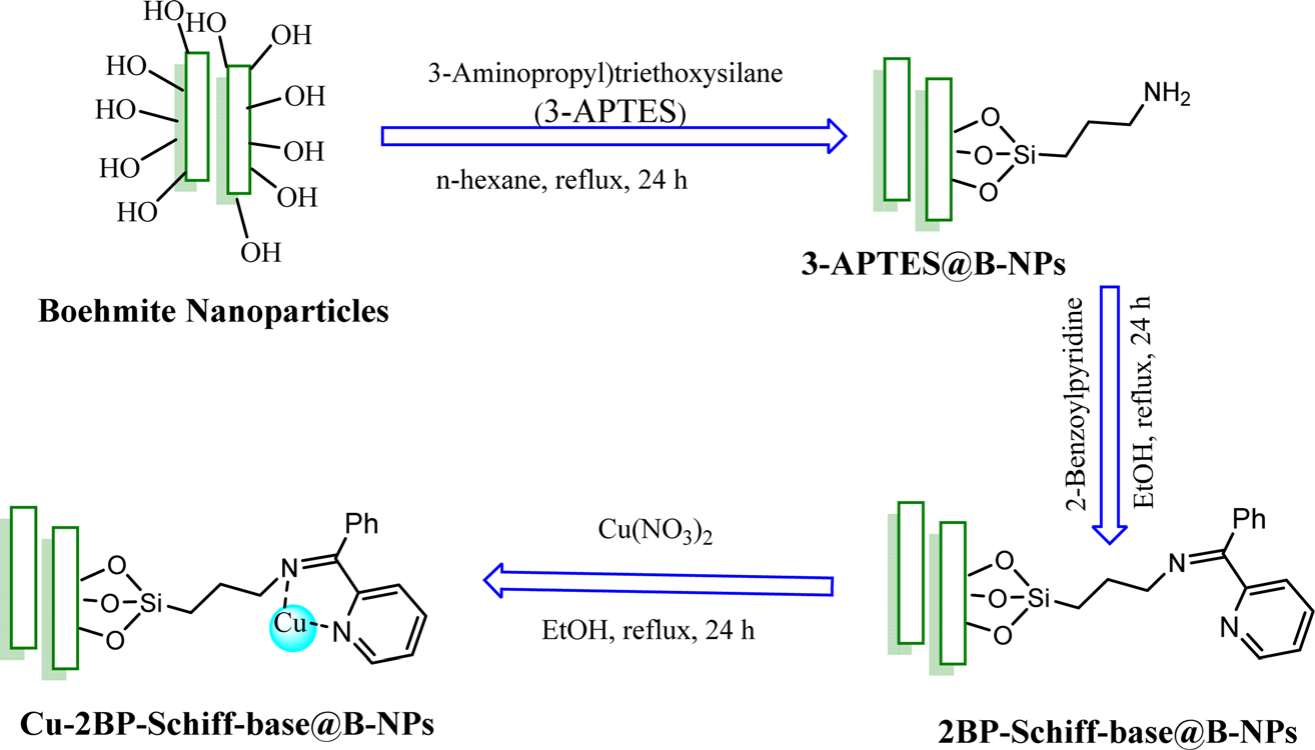
Synthesis of Cu-2BP-Schiff-base@B-NPs

3-APTES@B-NPs was synthesized from modification of B-NPs with 3-(aminopropyl)triethoxysilane (3-APTES) using a straightforward strategy. Briefly, 1 g of B-NPs with 1.5 mL of 3-APTES were poured into the flask and refluxed in *n*-hexane for 24 hours. After that, the powder was filtered and died at r.t. (Scheme 1).

In the next step, 1 g of 3-APTES@B-NPs were refluxed with 1 mmol of 2-benzoylpyridine (2BP) ligand in ethanol for 24 hours so that the amine reacts with the ketone carbonyl group and supported Schiff-base was formed (2BP-Schiff-base@B-NPs). The conversion yield was found to be approximately 85%. The obtained 2BP-Schiff-base@B-NPs were isolated by simple filtration and washing with ethanol. Finally, 1 g of 2BP-Schiff-base@B-NPs were mixed with 1 mmol of Cu(NO_3_)_2_ under reflux condition of ethanol for 24 hours when Cu-2BP-Schiff-base@B-NPs were formed. The obtained Cu-2BP-Schiff-base@B-NPs were isolated by simple filtration and washed with DI-H_2_O and ethanol. The conversion yield was found to be approximately 95% (Scheme 1).

The formation of boehmite NPs was confirmed by FT-IR, XRD, BET and SEM techniques.

### 2.2. Synthesis of various 5-substituted tetrazole derivatives catalyzed by Cu-2BP-Schiff-base@B-NPs

A mixture of NaN_3_ (1.2 mmol) and arylnitrile (1 mmol), Cu-2BP-Schiff-base@B-NPs (25 mg) and 1 mL of PEG-400 (polyethylene glycol-400) was prepared in a glass tube, which was then sealed and stirred at 120 °C for a specific duration (Table 2). The reaction progress was monitored using TLC, and once the reaction was complete, the catalyst was filtered off and washed with ethyl acetate and HCl (4 N). The pure products were extracted from water by ethyl acetate and purified using thin-layer chromatography (in a mixture of *n*-hexane : ethyl acetate in a ratio of 8 : 2 as eluent).

### 2.3. Selected spectral data

#### 2.3.1 5-(3-Nitrophenyl)-1*H*-tetrazole

^1^H NMR (400 MHz, DMSO-d6): *δ*_H_ = 8.81 (s, 1H), 8.46–8.43 (d, *J* = 12 Hz, 1H), 8.40–8.37 (d, *J* = 12 Hz, 1H), 7.91–7.84 (t, *J* = 12 Hz, 1H) ppm.

#### 2.3.2 2-(1*H*-Tetrazol-5-yl)benzonitrile

^1^H NMR (400 MHz, DMSO-d6): *δ*_H_ = 8.10–8.05 (m, 2H), 7.95–7.89 (t, *J* = 12 Hz, 1H), 7.80–7.74 (t, *J* = 12 Hz, 1H) ppm.

#### 2.3.3 2-(1*H*-Tetrazol-5-yl)phenol

^1^H NMR (400 MHz, DMSO-d6): *δ*_H_ = 7.98–7.95 (d, *J* = 12 Hz, 1H), 7.42–7.36 (t, *J* = 12 Hz, 1H), 7.07–6.95 (m, 2H), 3.37 (br, 1H) ppm.

## 3. Results and discussion

**Fig. 1:**
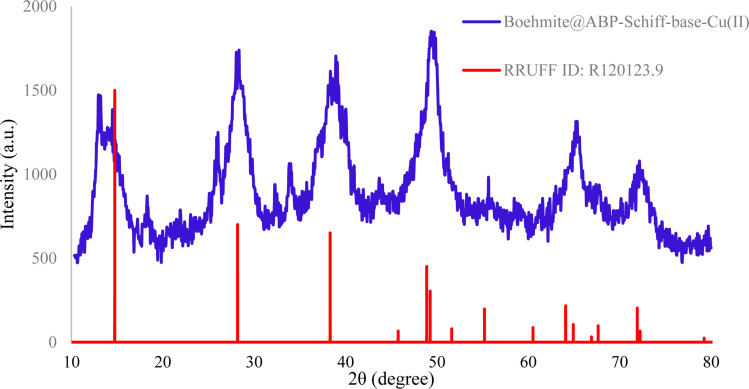
XRD patterns of Cu-2BP-Schiff-base@B-NPs.

To study the crystallinity of Cu-2BP-Schiff-base@B-NPs, the XRD technique was employed, and the results are shown in Fig. 1. The XRD pattern displays several 2*θ* peaks at 14.34 (0 2 0), 28.09 (1 2 0), 39.09° (0 3 1), 44.04° (1 3 1), 49.49° (0 5 1), 53.49° (2 0 0), 55.64° (1 5 1), 58.89° (0 8 0), 63.54° (2 3 1), 65.09° (0 0 2), 67.84° (1 7 1), and 72.49° (2 5 1), confirming the orthorhombic structure of boehmite (according to JCPDS No.: 01-072-0359). The crystallographic phase observed using XRD (Fig. 1) is similar to the boehmite phase (RRUFF ID: R120123.9), confirming the successful synthesis of boehmite and also crystallized in the orthorhombic crystal system.^42,43^ The size of the crystallite was estimated to be about 18 ± 2 nm according to Scherer's equation *D* = *Kλ*/(*β* cos *θ*).

**Fig. 2:**
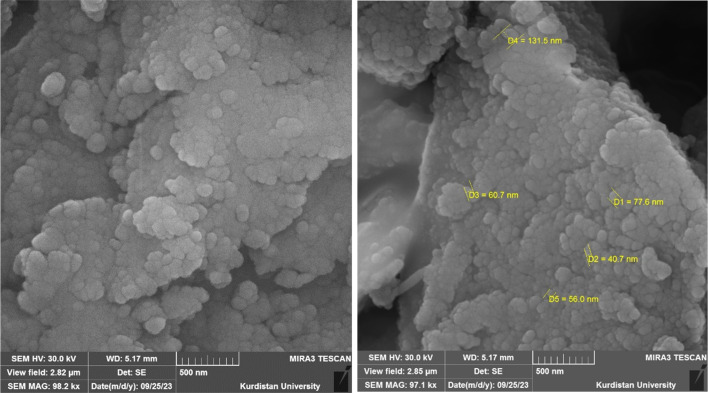
SEM images of Cu-2BP-Schiff-base@B-NPs.

SEM images of Cu-2BP-Schiff-base@B-NPs are presented in Fig. 2. The nanoparticles analyzed through FESEM exhibit a consistent semi-spherical shape and sizes ranging from 20–50 nm.

**Fig. 3:**
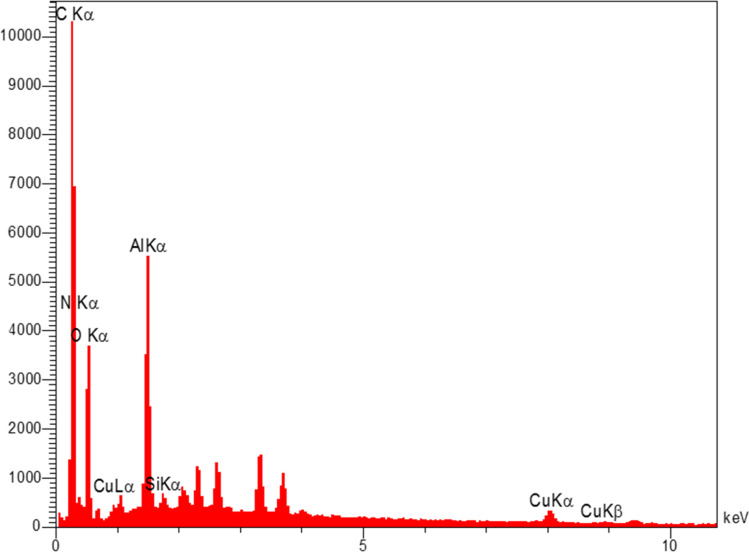
EDS data of Cu-2BP-Schiff-base@B-NPs.

**Fig. 4:**
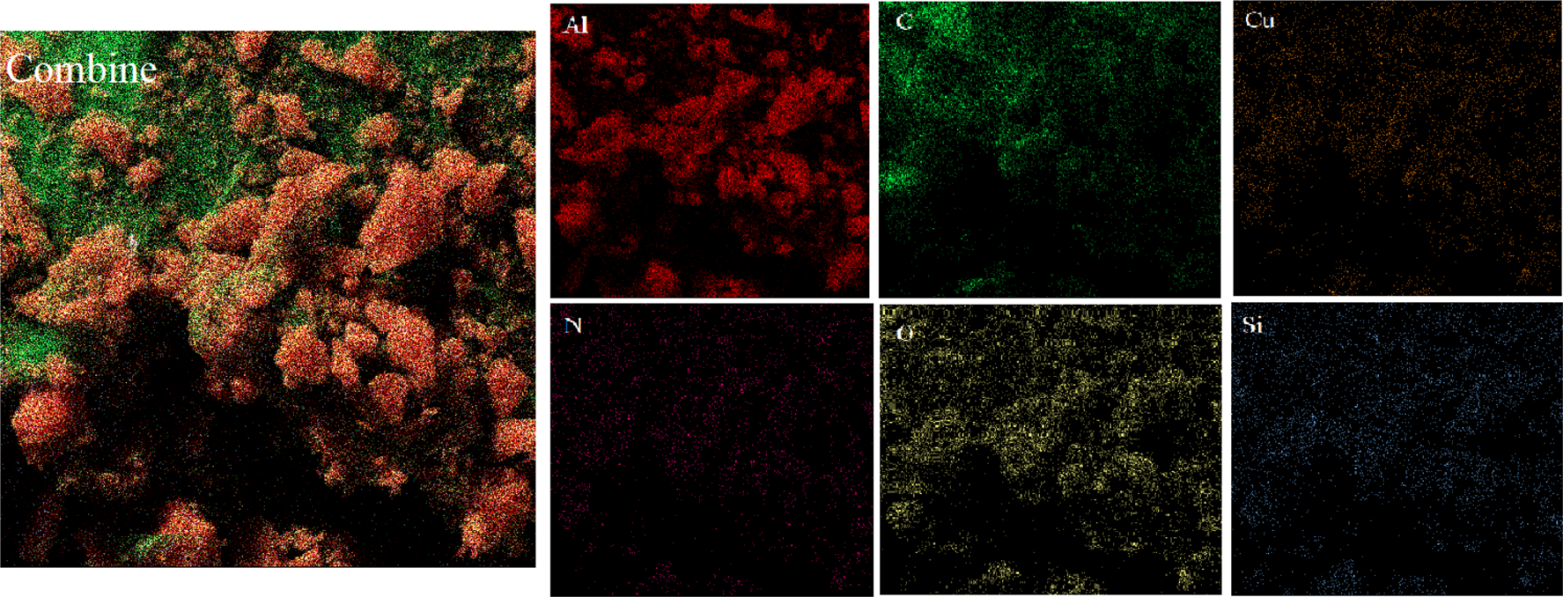
Elemental mapping of Cu-2BP-Schiff-base@B-NPs.

The elemental composition of Cu-2BP-Schiff-base@B-NPs was evaluated using energy-dispersive X-ray spectroscopy (EDX). The results in Fig. 3 show the presence of C, N, O, Si, Cu and Al elements in the catalyst structure, in which Cu confirmed the presence of a metal complex on boehmite. EDX elemental mapping images (Fig. 4) also showed a uniform distribution of these elements in the catalyst. The non-removable residue of approximately 13% was attributed to the formation of aluminum oxide. These findings were supported by AAC analysis, which revealed that 0.38 mmol of Cu was anchored on 1 g of nanocatalyst.

**Fig. 5:**
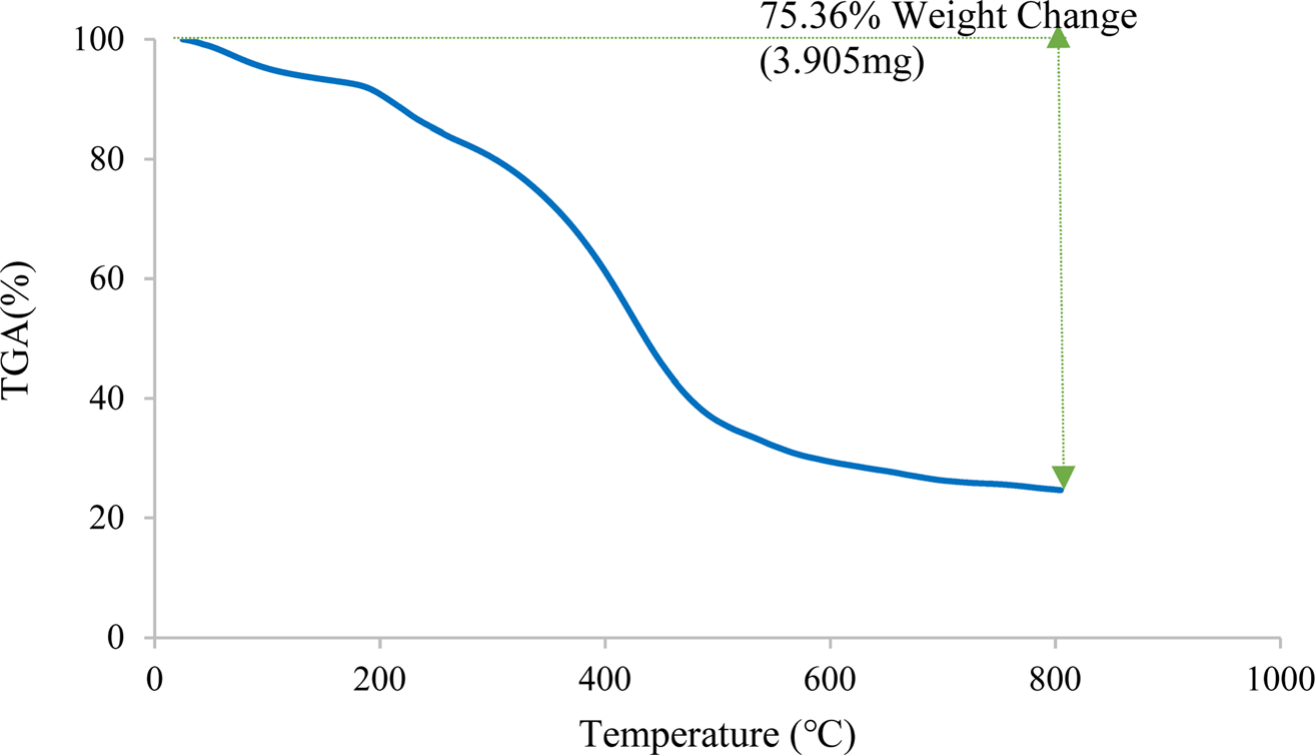
TGA analysis of Cu-2BP-Schiff-base@B-NPs.

Fig. 5 illustrates the thermal behavior of Cu-2BP-Schiff-base@B-NPs, indicating a significant decrease in weight percentage at around 153 °C due to the desorption of the solvent and water molecules from the catalyst surface.^44,45^ This was determined to be 6.8% based on TGA analysis. Another exothermic weight loss of 12.8% was observed above 198 °C, followed by a significant weight loss of 18% at 398 °C, which continued to approximately 500 °C, which is related to the decomposition of organic Schiff-base organic compounds on the surface of boehmite nanoparticles. Except for the evaporation of the solvent, no weight loss was observed in TGA analysis, therefore Cu-2BP-Schiff-base@B-NPs were stable up to 198 °C, at least.

**Fig. 6:**
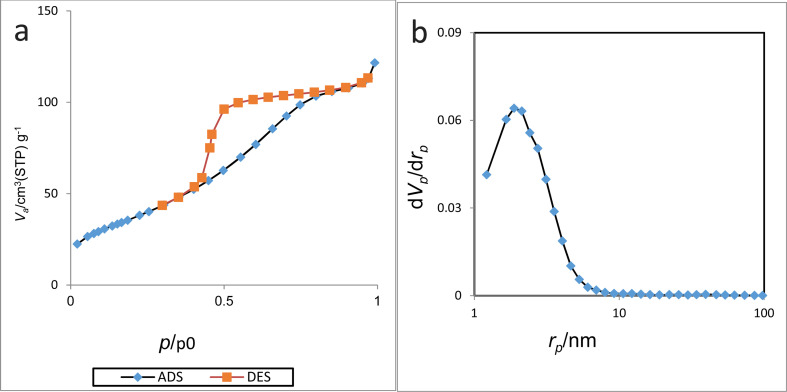
N_2_ adsorption–desorption isotherms (a) and BJH-Plot (b) of Cu-2BP-Schiff-base@B-NPs.

BET analysis was performed to determine the isotherm type, mean pore diameter, surface area, and total pore volume of Cu-2BP-Schiff-base@B-NPs. The nitrogen adsorption–desorption and nitrogen adsorption isotherm diagrams are shown in Fig. 6, which show that the surface area, total pore volume and mean pore diameter are 144.22 cm^3^ g^−1^, 0.18 m^2^ g^−1^, and 5.20 nm respectively. Furthermore, the isotherm was classified as a type IV isotherm from the IUPAC classification and fell under the mesoporous category.

**Fig. 7:**
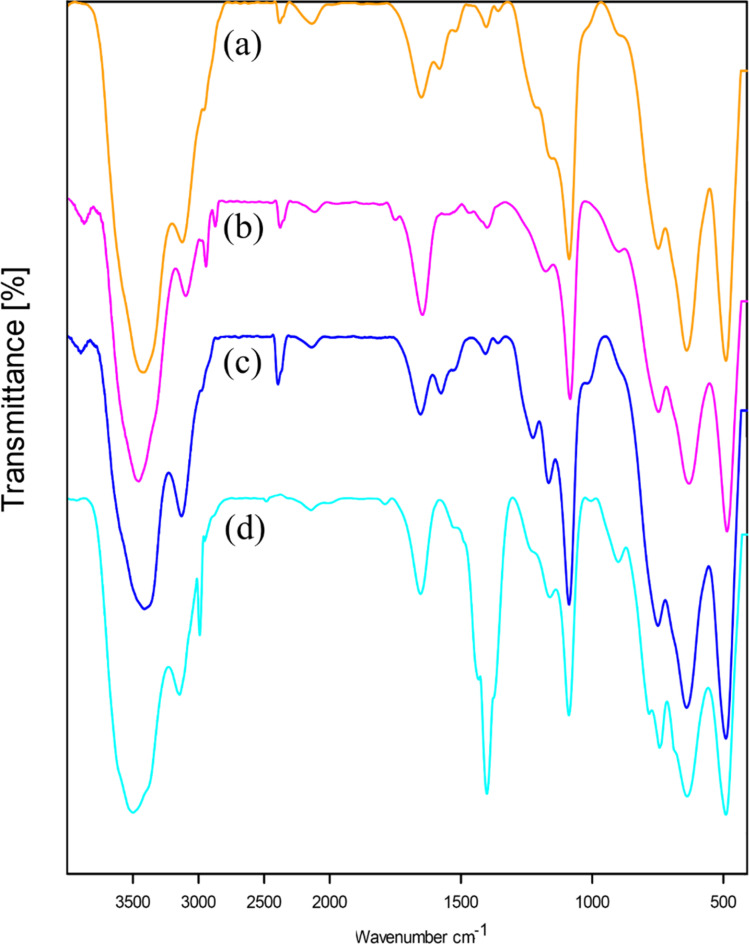
FT-IR spectra of (a) boehmite, (b) 3-APTES@B-NPs, (c) 2BP-Schiff-base@B-NPs, and (d) Cu-2BP-Schiff-base@B-NPs.

FT-IR spectra of boehmite NPs (a), 3-APTES@B-NPs (b), 2BP-Schiff-base@B-NPs (c), and Cu-2BP-Schiff-base@B-NPs (d) are displayed in Fig. 7. In the boehmite nanoparticles (Fig. 7a), strong bands were observed at 3392 and 3102 cm^−1^ corresponding to asymmetric and symmetric O–H stretching vibrations. The absorption peaks at 737, 629, and 479 cm^−1^ are due to Al–O stretching vibrations.^46^ In this spectrum, the peak of 1076 cm^−1^ is due to the presence of hydrogen bonds (OH⋯OH). It should be noted that the vibration of the nitrate impurity was indicated at 1630 cm^−1^. The attachment of (3-aminopropyl)-triethoxysilane to the surface of boehmite nanoparticles was confirmed by observing the characteristic bending and stretching vibrations of C–H and Si–O bonds at 2926–2856, and 1075 cm^−1^, respectively, as depicted in Fig. 7b–d. Moreover, the band at 1638 cm^−1^ in the spectrum of 2BP-Schiff-base@B-NPs (Fig. 7c) was assigned to the C=N (imine) stretching vibration, indicating covalent functionalization of boehmite nanoparticles with the 2-benzoylpyridine ligand through a condensation reaction between the –NH_2_ group with 2-benzoylpyridine on the surface of boehmite nanoparticles. Subsequent complexation with copper resulted in a shift of the C=N band to a lower wavenumber (1633 cm^−1^) in the spectrum of Cu-2BP-Schiff-base@B-NPs, suggesting the coordination between the copper and nitrogen atom of the ligand (Fig. 7d).

### 3.1. Catalytic studies

The catalytic activity of Cu-2BP-Schiff-base@B-NPs and their effect on some chemical reactions was investigated in the preparation of 5-substituted 1*H*-tetrazole derivatives.

In order to optimize the reaction conditions, benzonitrile and sodium azide (NaN_3_) were used for the synthesis of 5-substituted-1*H*-tetrazole and this process was investigated under different conditions such as the amounts of Cu-2BP-Schiff-base@B-NPs, temperature, and solvent (Table 1). In the beginning, the amount of Cu-2BP-Schiff-base@B-NPs was checked. First, 30 mg of catalyst was used, and the reaction yield was 98%. Then, 25, 20, and 15 mg of Cu-2BP-Schiff-base@B-NPs were also checked. By changing the amount of catalyst from 20 to 15 mg, the reaction yield decreased, therefore, 25 mg for Cu-2BP-Schiff-base@B-NPs was considered as the suitable amount of Cu-2BP-Schiff-base@B-NPs for this reaction. In the study of the solvent effect, PEG-400 was considered the best solvent, because this reaction was also checked in DMSO, H_2_O, EtOH, *n*-hexane as the solvent in which PEG-400 showed the best outcome (Table 1, entry 3). The reaction resulted in a low yield in aprotic polar and nonpolar solvents. However, polar protic solvents showed a higher yield of the tetrazoles. The temperature change was also checked, and the most favorable temperature was 120 °C.

**Table 1:** Evaluation of the reaction parameter on the synthesis of 5-phenyl-1*H*-tetrazole over the catalysis of Cu-2BP-Schiff-base@B-NP

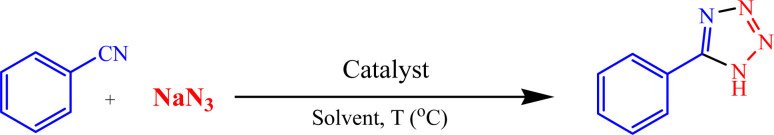						
Entry	Catalyst	Catalyst (mg)	Solvent	Temperature (°C)	Time (min)	Yield^a^ (%)
1	—	—	PEG-400	120	240	N.R
2	Boehmite	40	PEG-400	120	120	Trace
3	Cu-2BP-Schiff-base@B-NPs	20	PEG-400	120	200	60
4	Cu-2BP-Schiff-base@B-NPs	25	PEG-400	120	120	95
5	Cu-2BP-Schiff-base@B-NPs	30	PEG-400	120	120	95
6	Cu-2BP-Schiff-base@B-NPs	40	PEG-400	120	120	96
7	Cu-2BP-Schiff-base@B-NPs	25	H_2_O	Reflux	240	N.R
8	Cu-2BP-Schiff-base@B-NPs	25	EtOH	Reflux	240	20
9	Cu-2BP-Schiff-base@B-NPs	25	DMSO	120	240	50
10	Cu-2BP-Schiff-base@B-NPs	25	*n*-Hexan	Reflux	240	N.R
11	Cu-2BP-Schiff-base@B-NPs	25	Ethylene glycol	120	210	59
12	Cu-2BP-Schiff-base@B-NPs	25	PEG-400	100	170	N.R

a Isolated yield.

To evaluate the potential of Cu-2BP-Schiff-base@B-NPs in the synthesis of tetrazoles, different benzonitriles were investigated. Aryl nitriles with electron-withdrawing substituents and electron-donating substituents were evaluated and corresponding tetrazoles were formed in high yields and high TOF values. Also, heterocyclic aromatic nitriles were investigated. For example, 2-pyridinecarbonitrile was used and 2-(1*H*-tetrazol-5-yl)pyridine product was synthesized with 86% yield within 4 h. Unfortunately, no acceptable results were observed for the synthesis of tetrazole derivatives resulting from the [3 + 2] cycloaddition reaction of aliphatic nitriles with sodium azide.

Cu-2BP-Schiff-base@B-NPs showed good selectivity for dicyano-functionalized benzonitriles such as phthalonitrile (Table 2, entry 5) for the synthesis of the corresponding tetrazoles (Scheme S1, ESI†). These findings emphasize the potential of using a catalyst in organic synthesis to increase efficiency and selectivity.

**Table 2:** Synthesis of 5-substituted 1*H*-tetrazole derivatives catalyzed by Cu-2BP-Schiff-base@B-NPs

Entry	Nitrile	Time (min)	Yield^a^ (%)
1	4-Nitrobenzonitrile	120	81
2	3-Nitrobenzonitrile	20	91
3	2-Hydroxybenzonitrile	25	90
4	2-Pyridinecarbonitrile	240	82
5	Phthalonitrile	20	91
6	Benzonitrile	120	95
7	2-Chlorobenzonitrile	30	78
8	4-Chlorobenzonitrile	120	81
9	4-Bromobenzonitrile	180	86
10	*p*-Tolunitrile	130	95
11	2-Fluorobenzonitrile	240	76
12	3-(Trifluoromethyl)benzonitrile	20	93

a Isolated yield.

**Scheme 2:**
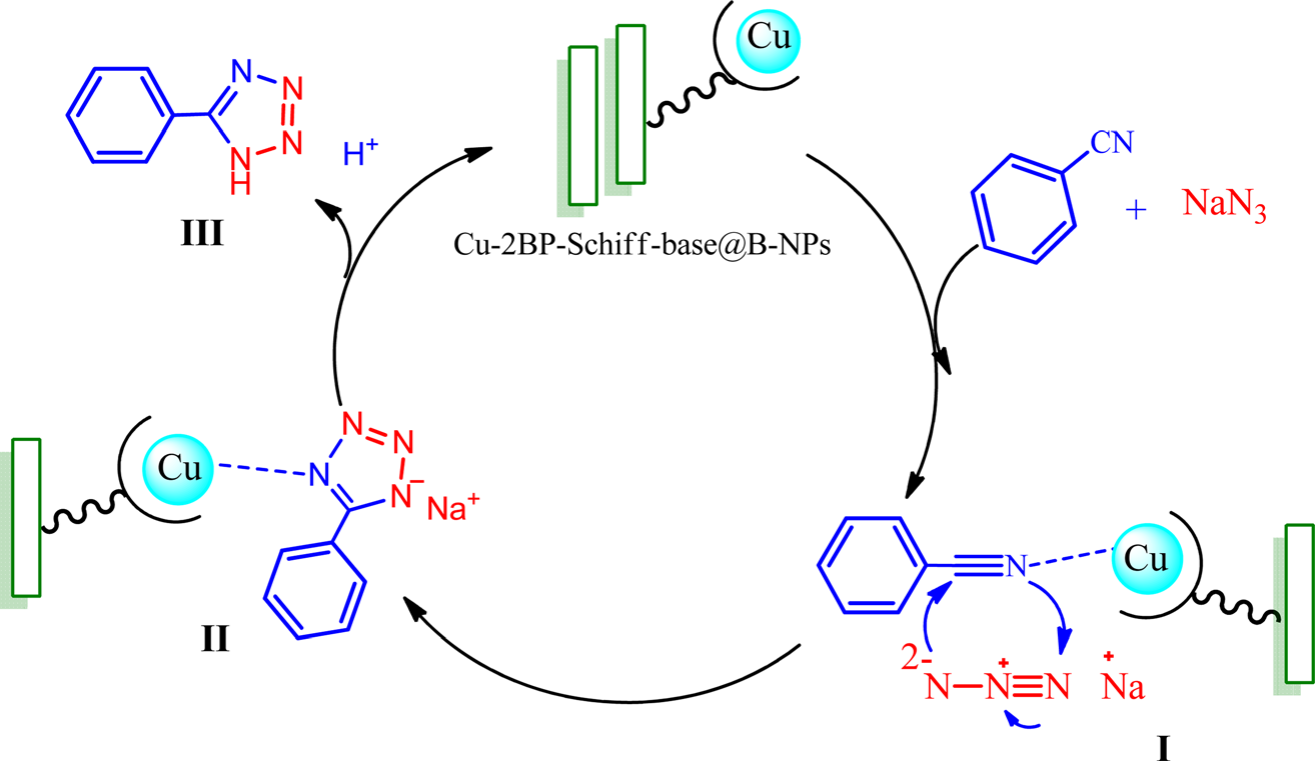
A suggested mechanism for the synthesis of tetrazoles in the presence of Cu-2BP-Schiff-base@B-NPs.

Scheme 2 outlines a suggested mechanism for the synthesis of tetrazole-catalyzed Cu-2BP-Schiff-base@B-NPs, based on previous reports.^47–49^ The process begins with the approach of the nitrile to the Cu complex, resulting in the intermediate (I). Subsequently, the azide ion undergoes a [3 + 2] cycloaddition with the activated nitrile bond, forming an intermediate (II) that is readily produced. Finally, the corresponding 5-aryl-1*H*-tetrazole product is obtained by protonating the anionic intermediate and acidifying the reaction using the HCl solution.

**Fig. 8:**
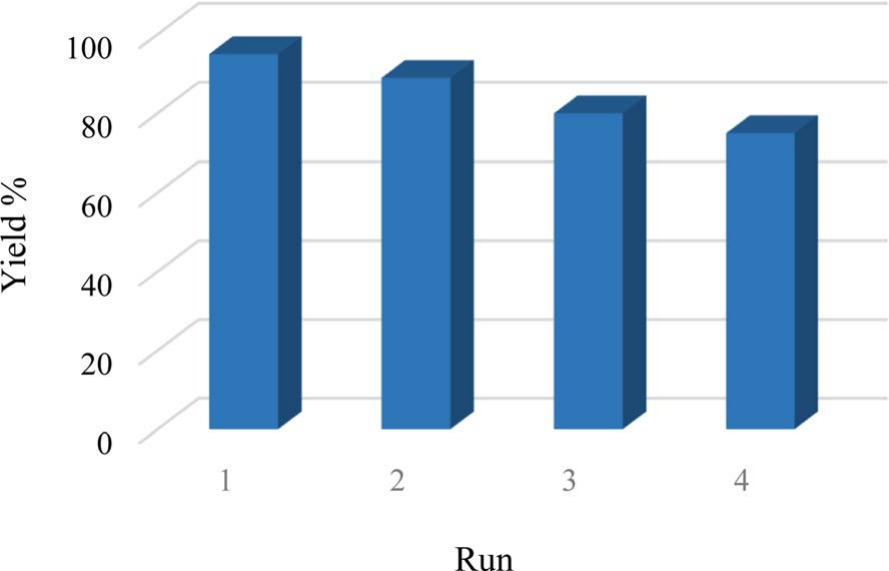
Reusability of Cu-2BP-Schiff-base@B-NPs in the model reaction.

One of the most notable features of environmentally friendly catalysts is their ability to be recycled, distinguishing them from conventional catalysts. In this regard, a study was conducted to evaluate Cu-2BP-Schiff-base@B-NPs's ability to be recycled in a model reaction. After the reaction, the catalyst was separated by simple filtration, washed with HCl solution (4 N) and ethanol, and reused in subsequent cycles (Fig. 8). Fig. 8 demonstrates that Cu-2BP-Schiff-base@B-NPs can be recovered and reused for up to four cycles without a significant loss of the catalytic activity or performance degradation.

Hot filtration was conducted on the model reaction. After 50 min (half the reaction time), heating was stopped, then the nanocatalyst was removed. The filtered reaction was reintroduced into the oil bath and allowed to react for another 50 min, but the product yield did not increase significantly (about 43%). These results indicate the heterogeneous nature of this catalyst.

The catalytic efficiency of Cu-2BP-Schiff-base@B-NPs was compared with other transition metal-containing catalysts that are reported in the literature for the synthesis of 5-aryl-1*H*-tetrazole. The comparative overview revealed that Cu-2BP-Schiff-base@B-NPs exhibited one of the highest reported yields. It is noteworthy that Cu-2BP-Schiff-base@B-NPs required less reaction time and produced excellent yields compared to other approaches listed in Table S1 (ESI).† Furthermore, 5-substituted tetrazoles were formed in PEG-400 as a green solvent.

## 4. Conclusions

In summary, we successfully prepared Boehmite nanoparticles using inexpensive and readily available starting materials *via* the coprecipitation method. The X-ray diffraction patterns show that the sample has an orthorhombic structure, and SEM images show particles with a regular shape. In addition, a new copper-Schiff-base organometallic complex by immobilizing it onto boehmite (Cu-2BP-Schiff-base@B-NPs) was developed. The synthesized heterogeneous catalyst was characterized using various analytical techniques, including TGA, FTIR, XRD, SEM, AAS, BET, and MAP. These techniques confirmed that the organocatalyst was grafted onto the surface of the NPs. The catalytic effect of Cu-2BP-Schiff-base@B-NPs nanocatalyst was evaluated as a powerful catalyst for the click reaction between aryl nitriles and NaN_3_ in PEG-400 as an environmentally friendly solvent. This method successfully produced the desired heterocycles using different benzaldehyde derivatives with electron-withdrawing and donating groups. This approach is cost-effective, stable, scalable, and reusable up to five times, providing metal-free catalysis and ensuring a safe process.

## Conflicts of interest

There are no conflicts to declare.

## Supplementary Material

RA-014-D4RA03139C-s001
