# Supplementary material for: A new Schiff base 2-benzoylpyridine-based copper complex on boehmite nanoparticles as a recoverable nanocatalyst for the homoselective synthesis of 5-substituted tetrazoles
Source: RSC Adv. 2024 May 20;14(23):16269–77. doi: 10.1039/d4ra03139c (PMC11103565; doi:10.1039/d4ra03139c)
Supplement: RA-014-D4RA03139C-s001 [file RA-014-D4RA03139C-s001.pdf]

Electronic Supplementary Material (ESI) for RSC Advances.
This journal is © The Royal Society of Chemistry 2024

# A new Schiff-base 2-benzoylpyridine Complex Copper on boehmite nanoparticles as a recoverable catalyst for the homoselective synthesis of 5-substituted tetrazoles

**Elham Mohseni[1], Arash Ghorbani-Choghamarani*[2], Bahman Tahmasbi*[1], Masoomeh Norouzi[1]**

*[1]Department of Chemistry, Faculty of Science, Ilam University, P. O. Box 69315516, Ilam, Iran.*

*[2]Department of Organic Chemistry, Faculty of Chemistry, Bu-Ali Sina University, Hamedan, 6517838683, Iran. E-mail: b.tahmasbi@ilam.ac.ir, a.ghorbani@basu.ac.ir*

## Abstract

In this research, boehmite nanoparticles (B-NPs) were prepared by a simple progress and then its surface was modified by 3-aminopropyl)triethoxysilane (3-APTES). The modified B-NPs (3-APTES@B-NPs) were functionalized by 2-benzoylpyridine Schiff-base ligand toward immobilization of Schiff-base 2-benzoylpyridine ligand on the 3-APTES@B-NPs's surface (2BP-Schiff-base@B-NPs). Finally, copper ions were coordinated with supported Schiff-base ligand on B-NPs toward formation of final catalyst (Cu-2BP-Schiff-base@B-NPs). The prepared Cu-2BP-Schiff-base@B-NPs was characterized by FT-IR, BET, XRD, SEM, AAS, TGA, EDX and mapping. In the next part, Cu-2BP-Schiff-base@B-NPs was applied as a homoselective and recyclable catalyst for the synthesis of a diverse range of 5-substituted tetrazoles in PEG-400 as a green solvent. The main benefits of this protocol are high homoselectivity attribute, short reaction times, high product yields and TOF values, and more addition the catalyst's ability to be recycled at least four times without significant losing catalytic efficiency.

**Keywords:** Schiff-base ligand, copper complex; boehmite nanoparticles, homoselective catalyst, 5-substituted tetrazoles

$^1$H NMR spectral data

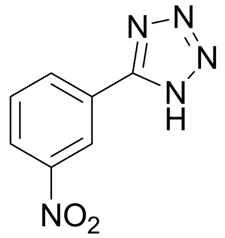

**5-(3-nitrophenyl)-1H-tetrazole**

$^1$H NMR (400 MHz, DMSO-d6): $\delta_H$= 8.81 (s, 1H), 8.46-8.43 (d, *J*= 12 Hz, 1H), 8.40-8.37 (d, *J*= 12 Hz, 1H), 7.91-7.84 (t, *J*= 12 Hz, 1H) ppm.

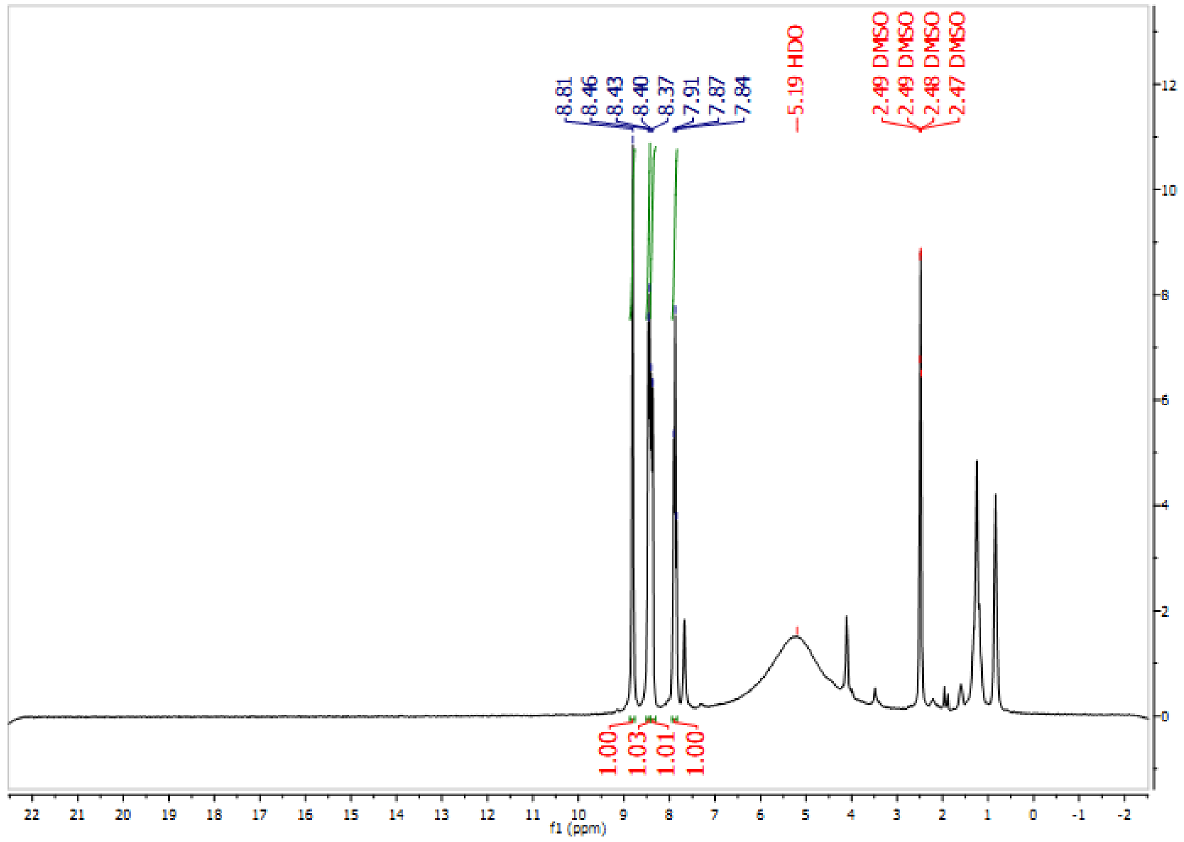

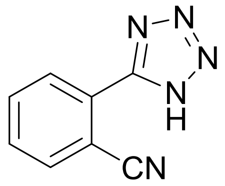


**2-(1H-tetrazol-5-yl)benzonitrile**

$^{1}$H NMR (400 MHz, DMSO-d6): $\delta_H$= 8.10-8.05 (m, 2H), 7.95-7.89 (t, *J*= 12 Hz, 1H), 7.80-7.74 (t, *J*= 12 Hz, 1H) ppm.

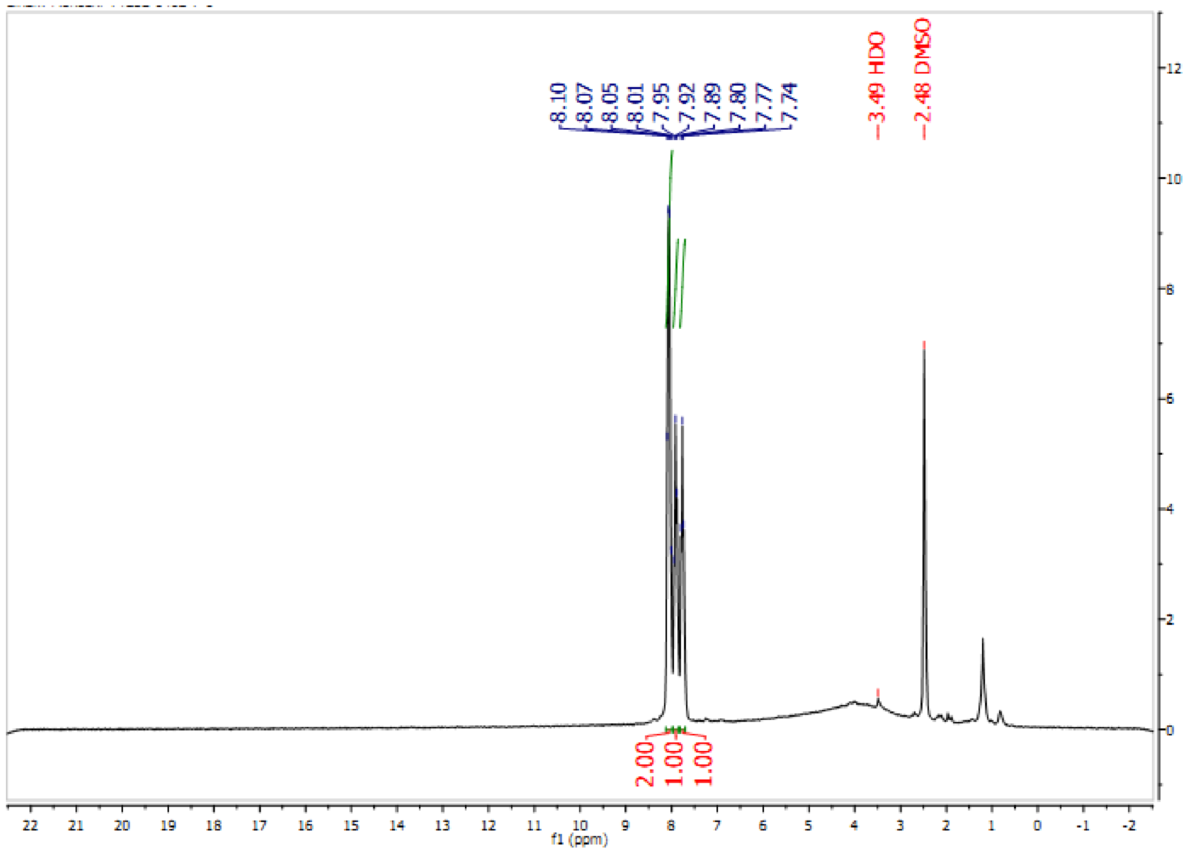

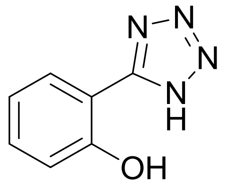

**2-(1H-tetrazol-5-yl)phenol**

$^1$H NMR (400 MHz, DMSO-d6): $\delta_H$= 7.98-7.95 (d, *J*= 12 Hz, 1H), 7.42-7.36 (t, *J*= 12 Hz, 1H), 7.07-6.95 (m, 2H), 3.37 (br, 1H) ppm.

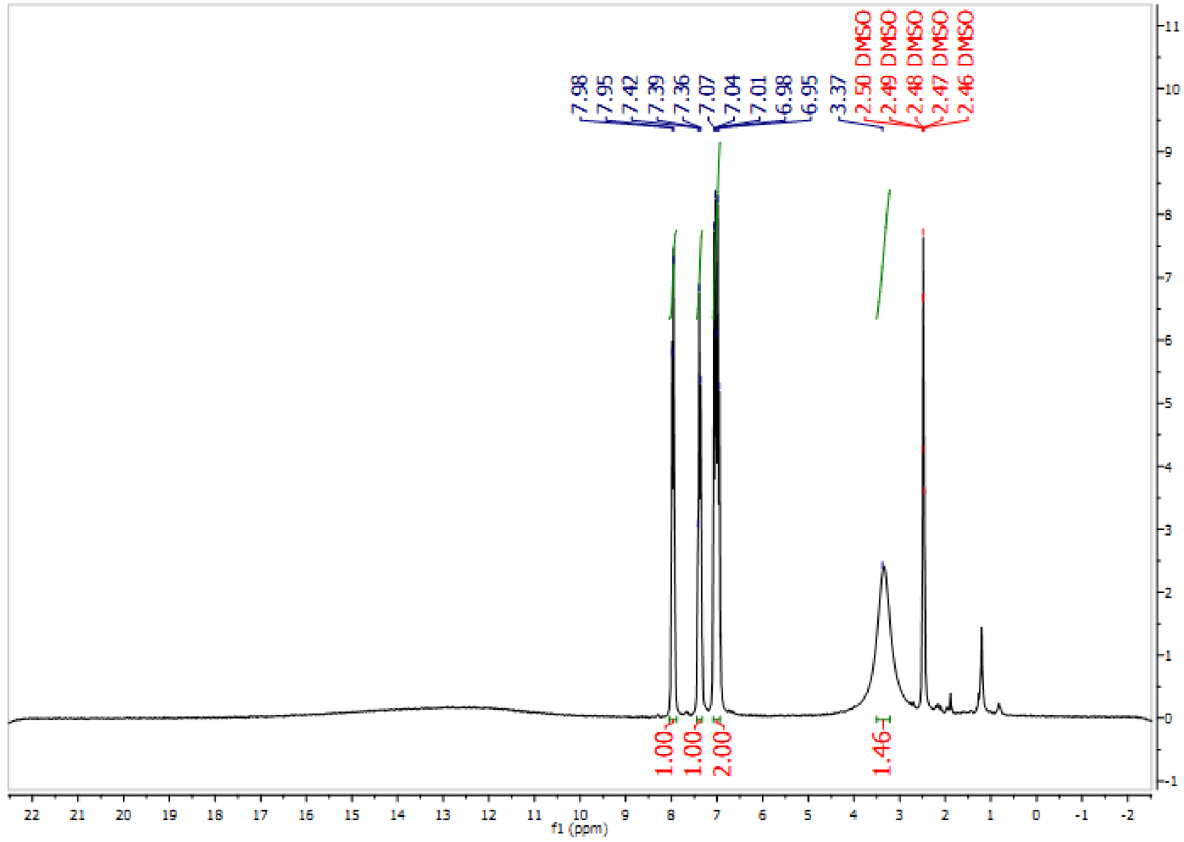

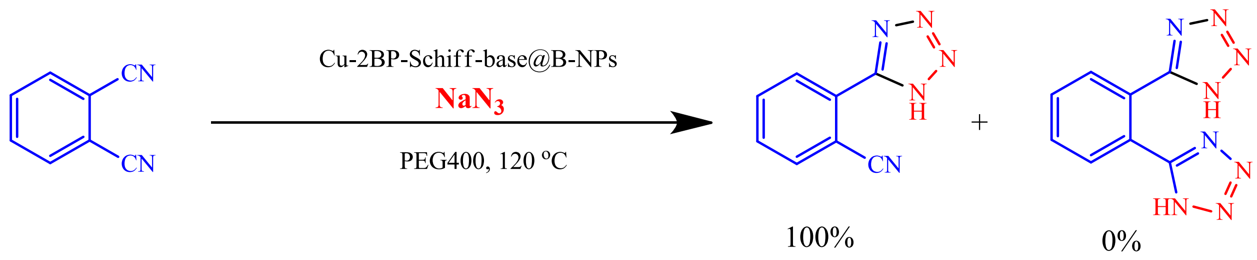


**Scheme S1.** Homoselectivity of Cu-2BP-Schiff-base@B-NPs in the synthesis of tetrazoles.

**Table S1.** Comparison of catalytic efficiency of Cu-2BP-Schiff-base@B-NPs in the model reaction of synthesis of 5-phenyl-1H-tetrazole.

| Entry | Catalyst | Time (h) | Yield (%) | Reference |
|---|---|---|---|---|
| 1 | CoY Zeolite | 14 | 95 | [1] |
| 2 | Cu–Zn alloy nanopowder | 10 | 94 | [2] |
| 3 | $B(C_6F_5)_3$ | 8 | 95 | [3] |
| 4 | $Fe_3O_4$@$SiO_2$/Salen Cu(II) | 7 | 81.1 | [4] |
| 5 | $Fe_3O_4$/ZnS HNSs | 24 | 86 | [5] |
| 6 | Mesoporous ZnS | 36 | 98 | [6] |
| 7 | $CuFe_2O_4$ | 12 | 90 | [7] |
| 8 | Nano ZnO/$Co_3O_4$ | 12 | 98 | [8] |
| 9 | $Fe_3O_4$@boehmite NPs | 4 | 97 | [9] |
| 10 | Ni-MP$(AMP)_2$@Fe-biochar | 4 | 97 | [10] |
| 11 | Cu-2BP-Schiff-base@B-NPs | 2 | 95 | This work |

[a]Isolated yield

## References


1. V. Rama, K. Kanagaraj and K. Pitchumani, *The Journal of Organic Chemistry*, 2011, **76**, 9090-9095.
2. G. Aridoss and K. K. Laali, *Journal*, 2011.
3. S. K. Prajapti, A. Nagarsenkar and B. N. Babu, *Tetrahedron Letters*, 2014, **55**, 3507-3510.
4. F. Dehghani, A. R. Sardarian and M. Esmaeilpour, *Journal of Organometallic Chemistry*, 2013, **743**, 87-96.
5. G. Qi, W. Liu and Z. Bei, *Chinese Journal of Chemistry*, 2011, **29**, 131-134.
6. L. Lang, H. Zhou, M. Xue, X. Wang and Z. Xu, *Materials Letters*, 2013, **106**, 443-446.
7. B. Sreedhar, A. S. Kumar and D. Yada, *Tetrahedron letters*, 2011, **52**, 3565-3569.
8. S. M. Agawane and J. M. Nagarkar, *Catalysis Science & Technology*, 2012, **2**, 1324-1327.

9. P. Moradi, *RSC advances*, 2022, **12**, 33459-33468.
10. P. Moradi and M. Hajjami, *New Journal of Chemistry*, 2021, **45**, 2981-2994.
